# SrTiO_3_-SrVO_3_ Ceramics for Solid Oxide Fuel Cell Anodes: A Route from Oxidized Precursors

**DOI:** 10.3390/ma16247638

**Published:** 2023-12-14

**Authors:** Javier Macías, Jorge R. Frade, Aleksey A. Yaremchenko

**Affiliations:** CICECO—Aveiro Institute of Materials, Department of Materials and Ceramic Engineering, University of Aveiro, 3810-193 Aveiro, Portugal; javier.macias@bondalti.com (J.M.); jfrade@ua.pt (J.R.F.)

**Keywords:** perovskite, titanate, vanadate, composite, SOFC, anode, electrical conductivity

## Abstract

Perovskite-type Sr(Ti,V)O_3-δ_ ceramics are promising anode materials for natural gas- and biogas-fueled solid oxide fuel cells, but the instability of these phases under oxidizing conditions complicates their practical application. The present work explores approaches to the fabrication of strontium titanate-vanadate electrodes from oxidized precursors. Porous ceramics with the nominal composition SrTi_1−*y*_V*_y_*O*_z_* (*y* = 0.1–0.3) were prepared in air via a solid state reaction route. Thermal processing at temperatures not exceeding 1100 °C yielded composite ceramics comprising perovskite-type SrTiO_3_, pyrovanadate Sr_2_V_2_O_7_ and orthovanadate Sr_3_(VO_4_)_2_ phases, while increasing firing temperatures to 1250–1440 °C enabled the formation of SrTi_1−*y*_V*_y_*O_3_ perovskites. Vanadium was found to substitute into the titanium sublattice predominantly as V^4+^, even under oxidizing conditions at elevated temperatures. Both perovskite and composite oxidized ceramics exhibit moderate thermal expansion coefficients in air, 11.1–12.1 ppm/K at 30–1000 °C, and insignificant dimensional changes induced by reduction in a 10%H_2_-N_2_ atmosphere. The electrical conductivity of reduced perovskite samples remains comparatively low, ~10^−1^ S/cm at 900 °C, whereas the transformation of oxidized vanadate phases into high-conducting SrVO_3−δ_ perovskites upon reduction results in enhancement in conductivity, which reaches ~3 S/cm at 900 °C in porous composite ceramics with nominal composition SrTi_0.7_V_0.3_O*_z_*. The electrical performance of the composite is expected to be further improved by optimization of the processing route and microstructure to facilitate the reduction of the oxidized precursor and attain better percolation of the SrVO_3_ phase.

## 1. Introduction

Oxide phases derived from perovskite-type SrTiO_3_ and SrVO_3_ are considered potential alternatives to traditional Ni-YSZ (yttria-stabilized zirconia) anode materials used in hydrocarbon-fueled solid oxide fuel cells (SOFCs) [1,2,3,4,5,6]. Apart from issues related to redox instability and microstructural degradation due to the agglomeration of nickel particles with time [7,8], Ni-YSZ cermets suffer from coking and poisoning by ppm levels of H_2_S contaminant in fuels such as natural gas or biogas [1,9]. In contrast, SrTiO_3_- and SrVO_3_-based ceramics are reported to exhibit good resistance to carbon deposition and tolerance towards sulfur poisoning [10,11,12,13,14,15].

Ceramic anode components based on SrTiO_3_ and SrVO_3_ have their pros and cons. The perovskite-type SrTiO_3_ lattice exhibits remarkable redox and dimensional stability in a wide range of T-p(O_2_) conditions [2,16], which is essential for SOFC anode applications. Although the electrical conductivity of SrTiO_3_ under reducing conditions can be improved by donor-type substitutions into one or both sublattices, it remains moderate, ≤10 S/cm under typical anode operation conditions [17,18,19,20]. Furthermore, donor-doped strontium titanates prepared under oxidizing conditions demonstrate a long relaxation of electrical conductivity due to the slow reduction kinetics at SOFC operation temperatures [21,22,23]. The characteristics of SrVO_3−δ_ relevant to SOFC anode application are the opposite. This perovskite exhibits high metallic-like electronic conductivity under reducing conditions, ~1000 S/cm at 800 °C [24,25,26], and non-negligible oxygen deficiency in the lattice [26,27], which may imply reasonable oxygen-ionic contribution to the total conductivity and is favorable for electrochemical performance. At the same time, undoped SrVO_3−δ_ ceramics have an excessive thermal expansion coefficient (TEC, average 18–19 ppm/K at 100–1000 °C [26,28]) that complicates their thermomechanical compatibility with other SOFC components. Also, perovskite-like SrVO_3−δ_ is stable only under reducing conditions below p(O_2_)~10^−15^ and 10^−17^ atm at 900 and 800 °C, respectively [24,26,29]. To some extent, the stability domain of the perovskite phase can be expanded via suitable substitutions into strontium and/or vanadium sublattices [29,30]. 

SrTiO_3_ and SrVO_3−δ_ form a continuous series of perovskite-like solid solutions under reducing conditions [28,31,32]. Balancing fractions of titanium and vanadium cations in the B sublattice enables a reasonable compromise between phase stability and the level of electrical conductivity and also allows one to adjust the thermochemical expansion [28,33]. In particular, SrTi_0.5_V_0.5_O_3−δ_ perovskite ceramics with intermediate titanium and vanadium content showed electrical conductivity of ~20 S/cm at 900 °C combined with a phase stability domain extended up to p(O_2_) of at least 10^−11^ atm at 900 °C and average TEC of 15 ppm/K [28]. The material still undergoes oxidative decomposition into titanium- and vanadium-rich phases when heated in air. The re-reduction does not recover the initial perovskite phase but may induce even slightly higher electrical conductivity compared to initial values [28].

Due to phase stability issues, Sr(Ti,V)O_3_-based anodes may be prepared either under reducing conditions or under an inert atmosphere where oxidation kinetics are sluggish [28,33]. However, from a practical point of view, it would be of interest to fabricate anodes of solid oxide cells under an ambient atmosphere (air) with a subsequent in-situ reduction, similar to what is practiced in the case of Ni-YSZ cermets. Therefore, the goal of the present study is to evaluate the possibility of preparing Sr(Ti,V)O_3_ precursors under oxidizing conditions and to assess their redox behavior. The reported experimental studies of oxidized V-doped SrTiO_3_ are mainly focused on the preparation of powdered samples with small vanadium additions (≤5 mol.%) at relatively low temperatures and their characterization for photocatalytic applications [34,35,36,37]. Park et al. [38] prepared mesoporous SrTi_0.8_V_0.2_O_3_ thin films and studied their room-temperature thermoelectric properties. Mantry et al. [39] attempted the preparation of SrTi_1−*x*_V*_x_*O_3_ (*x* = 0.05–0.20) ceramics via the solid state reaction method with sintering at 1250 °C and reported that all samples comprised phase impurities including Sr_2_V_2_O_7_. 

Two approaches to the oxidized Sr(Ti,V)O_3_ precursors are comparatively explored in the present work: (i) synthesis of single-phase perovskite ceramics; and (ii) preparation of composites based on the SrTiO_3_ and Sr-V-O phases. The studies are focused on ceramic samples with moderate vanadium additions (Ti:V ratio from 9:1 to 7:3) and porous microstructure (to simulate porous electrode layers of solid oxide cells) and include the characterization of phase composition, electrical conductivity, thermal expansion and the corresponding changes induced by reduction.

## 2. Materials and Methods

Materials with the nominal composition SrTi_−*y*_V*_y_*O*_z_* (STV; *y* = 0.1, 0.2, 0.3) were prepared by solid state reaction route. SrCO_3_ (purity > 99.9%, Sigma Aldrich, St. Louis, MO, USA), TiO_2_ (>99.8%, Sigma Aldrich) and V_2_O_5_ (>99%, Fluka) were used as starting chemicals. The mixtures of reagents taken in appropriate proportions were preliminarily calcined in air at 500 °C/5 h and 600 °C/5 h, with intermediate regrinding, to promote the onset of Sr-V-O phases at temperatures below the melting point of V_2_O_5_ (T_melt_ = 681 °C). Then, the pre-calcined precursor mixtures were divided into two batches. 

The first batch of each composition was calcined in air at 900–1100 °C, with a stepwise increase in temperature in increments of 50 °C, duration of 5 h at each step, and regrinding between the steps. Then, the powders were ball-milled with ethanol at 150 rpm for 4 h using a Retsch S1 planetary mill (Retsch GmbH, Haan, Germany), nylon containers and Tosoh tetragonal zirconia balls. After milling and drying, the powders were compacted uniaxially into disk-shaped pellets and sintered in air at 1000 °C for 5 h. Hereafter, these samples are notated as STV-C or STV*yy*-C, where “C” stands for “composite” and “*yy*” indicates the percentage of vanadium cations in the B-sublattice.

The second batch of each composition was calcined in air in the regime of increasing calcination temperature stepwise, with steps of 40–100 °C and a duration of 5 h at each step, with regrinding between the steps, until X-ray diffraction (XRD) confirmed the absence of secondary phases. The highest calcination temperature was 1250, 1350 and 1440 °C for *y* = 0.1, 0.2 and 0.3, respectively. After subsequent ball-milling and drying, disc-shaped pellets were compacted and sintered in air at 1100 °C for 5 h. These samples are referred to as STV-P or STV*yy*-P, where “P” indicates “perovskite”. 

The sintered ceramic samples were polished using SiC grinding paper (Buehler, Leinfelden-Echterdingen, Germany). The density of the prepared ceramics was calculated using the geometric dimensions and mass of the polished samples. Rectangular bars for electrical and dilatometric measurements were cut out of the disk-shaped pellets using a Struers Minitom precision cutting machine (Struers, Copenhagen, Denmark) with a diamond cut-off wheel. Powdered samples for XRD studies and thermogravimetric analyses (TGAs) were prepared by grinding sintered ceramics in a mortar. 

The XRD patterns of the powdered samples were recorded on a PANalytical X’Pert PRO diffractometer (PANalytical, Almelo, The Netherlands, CuK_α_ radiation, step 0.026°) in the range 2θ = 20–80°. The lattice parameters were calculated from the XRD data using FullProf software (profile matching method). Microstructural characterization was performed by scanning electron microscopy (SEM) using a Hitachi SU-70 microscope (Hitachi, Tokyo, Japan) equipped with a Bruker Quantax 400 detector (Bruker, Berlin, Germany) for energy dispersive spectroscopy (EDS) analysis. Thermogravimetric analysis (TGA, Setaram SetSys 16/18 instrument (Setaram, Caluire, France); sensitivity: 0.4 µg; initial sample weight: ~0.5 g) was carried out on heating in a flowing 10%H_2_-N_2_ mixture in the temperature range of 25–1000 °C with a constant rate of 2 °C/min followed by the isothermal step at 1000 °C. Dilatometric studies were conducted using a vertical alumina Linseis L70 dilatometer (Linseis, Selb, Germany) on heating/cooling at 3 °C/min in flowing air or a 10%H_2_-N_2_ mixture. 

Total electrical conductivity (σ) was determined employing AC impedance spectroscopy (2-probe method, Agilent 4284A precision LCR meter (Agilent, Santa Rosa, CA, USA); frequency range: 20 Hz–1 MHz; AC amplitude 1 V) and bar-shaped ceramic samples with porous Pt electrodes (Heraeus CL-11–5349 platinum paste, sintering at 1000 °C for 30 min) applied onto the end-faces of the bars. The measurements were performed in air in the temperature range of 750–1000 °C in a stepwise cooling regime. The relaxation of the electrical conductivity of samples on reduction was studied isothermally at 900 °C as a function of time on switching from an oxidizing (air) to a reducing atmosphere. A common procedure for the reduction of traditional Ni-YSZ cermet anodes during solid oxide cell start-up is purging with diluted hydrogen (e.g., 5–10 vol.% in nitrogen) [40,41]. A similar reducing atmosphere, a 10%H_2_-N_2_ mixture, was employed in the present work. 

The values of oxygen partial pressure p(O_2_) in the gas flow during the experiments were monitored employing homemade potentiometric YSZ sensors. The representative p(O_2_) value in the 10%H_2_-N_2_ gas mixture corresponded to ~10^−20^ atm at 900 °C.

## 3. Results and Discussion

### 3.1. Phase Composition, Structure and Microstructure of As-Prepared Samples

**Figure 1:**
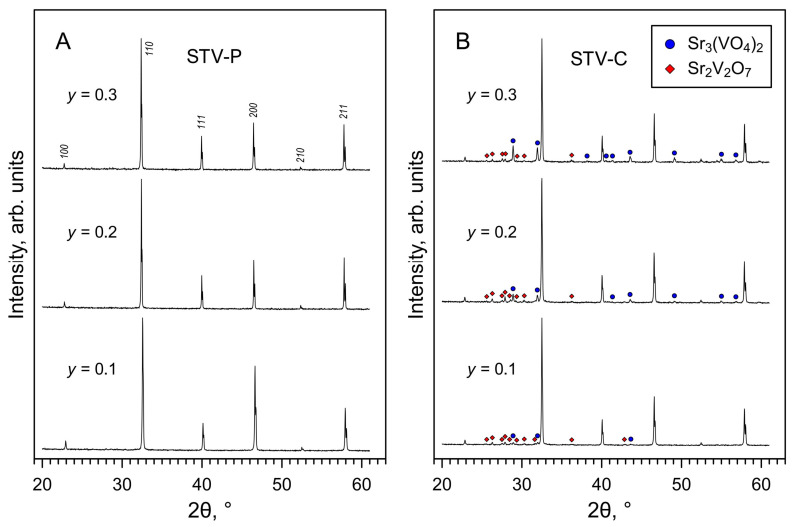
XRD patterns of as-prepared (**A**) STV-P and (**B**) STV-C samples. The reflections of the main perovskite phase are indexed in space group *Pm*$\bar{3}$*m*. The reflections of the Sr_2_V_2_O_7_ and Sr_3_(VO_4_)_2_ phases are marked according to ICDD PDF no. 01-071-1593 and 01-081-1844, respectively.

XRD analysis of the as-prepared STV-P samples confirmed the formation of a SrTiO_3_-based phase with a cubic perovskite structure (Figure 1A), without detectable phase impurities. All reflections in the XRD patterns were indexed in space group *Pm*$\bar{3}$*m*. The calculated lattice parameters decrease with increasing vanadium content in the perovskite lattice (Table 1), which is reasonable considering that the ionic radii of V^4+^ and V^5+^ cations are smaller than that of Ti^4+^ [42]. This trend is also in agreement with data reported for the SrTi_1−*y*_V*_y_*O_3−δ_ series prepared under reducing conditions [28,31,32].

Reduced SrTi_1−*y*_V*_y_*O_3−δ_ perovskites comprise vanadium cations in an average oxidation state of ≤4+ with a comparatively small fraction of V^3+^ [28]. Under oxidizing conditions, vanadium tends to a pentavalent state in simple V_2_O_5_ oxide [24,43] as well as in pseudo-binary compounds such as $Sr_{4}V_{2}^{5+}O_{9}$, $Sr_{3}{\left(V_{2}^{5+}O_{4}\right)}_{2}$, $Sr_{2}V_{2}^{5+}O_{7}$ and $Sr{\left(V^{5+}O_{3}\right)}_{2}$ in the SrO-V_2_O_5_ system [44,45,46]. This is also reflected by the ready transformation of perovskite-type $SrV^{4+}O_{3-\delta }$ into $Sr_{2}V_{2}^{5+}O_{7}$ pyrovanadate upon thermal treatment in air [25,26]. Hence, the oxidation state of vanadium cations in oxidized SrTi_1−*y*_V*_y_*O_3±δ_ is expected to be not less than 4+. It is commonly known that a close-packed cubic perovskite structure cannot accommodate interstitial oxygen ions. Taking the site conservation condition into account, the formation of oxidized SrTi_1−*y*_V*_y_*O_3±δ_ may occur according to one of the following two scenarios:

(a) High-temperature treatments force the reduction of vanadium cations and their incorporation into the titanium sublattice in the V^4+^ state. This should lead to the formation of oxygen-stoichiometric perovskite.
(b) A scenario similar to other oxidized donor-doped strontium titanates with a nominal cation stoichiometry such as Sr_1−*x*_La*_x_*TiO_3±δ_ or SrTi_1−*y*_Nb*_y_*O_3±δ_. Incorporation of a higher-valence cation into one of the sublattices, e.g., V^5+^ into the Ti^4+^ sublattice, is compensated by the formation of extended defects in the lattice—SrO shear planes characteristic of Ruddlesden-Popper phases combined with A-site cation vacancies, and/or defect clusters built of donor cations and interstitial oxygen ions [18,23,47,48].

Whatever the mechanism, the XRD results support the formation of phase-pure oxidized SrTi_1−*y*_V*_y_*O_3±δ_ perovskites. 

In the case of the STV-C series, the XRD results showed the formation of multiphase samples comprising at least two phases in addition to the main SrTiO_3_-based cubic perovskite (Figure 1B). These two phases were identified as rhombohedral strontium orthovanadate Sr_3_(VO_4_)_2_ and tetragonal strontium pyrovanadate Sr_2_V_2_O_7_. It is noteworthy that the latter is a low-temperature β-Sr_2_V_2_O_7_ modification reported by Baglio and Dann [49]. In a previous work [26], a solid state synthesis of strontium pyrovanadate and oxidation of perovskite-type SrVO_3−δ_ in air yielded a high-temperature triclinic α- Sr_2_V_2_O_7_ polymorph. Thus, the processing of STV-C samples at relatively low temperatures (compared to the STV-P series) prevented the formation of solid solutions and produced composite samples comprising strontium titanate and strontium vanadate phases. The fractions of vanadates, particularly Sr_3_(VO_4_)_2_, reasonably increase with increasing vanadium content in nominal SrTi_1−*y*_V*_y_*O*_z_* (Figure 1B).

**Figure 2:**
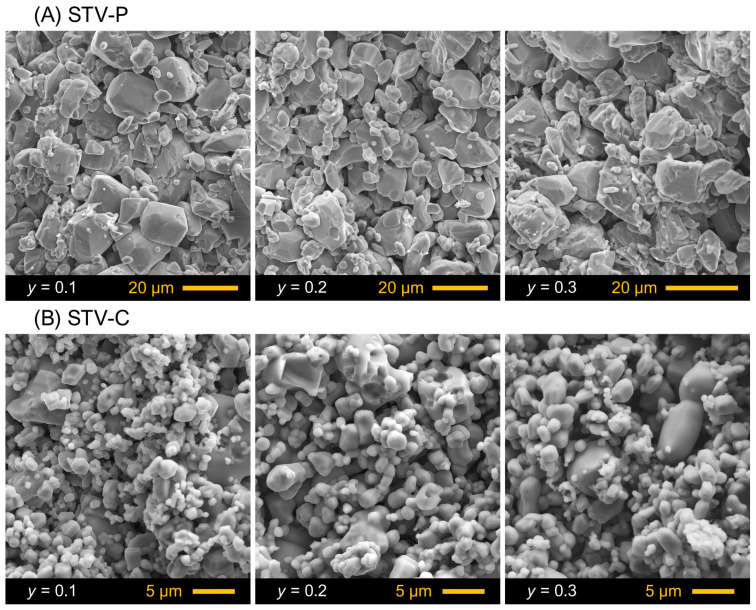
SEM images of fractured cross-sections of as-prepared (**A**) STV-P and (**B**) STV-C ceramics.

The prepared ceramics of both series were porous, as dictated by the low sintering temperatures (Figure 2). The estimations showed that the relative density of STV-P samples corresponded to 63–64% (Table 1). The grain size varied from 1.5–2.0 to 15–19 µm (Figure 2A). Changes in V content did not have a visible impact on the microstructure. 

**Figure 3:**
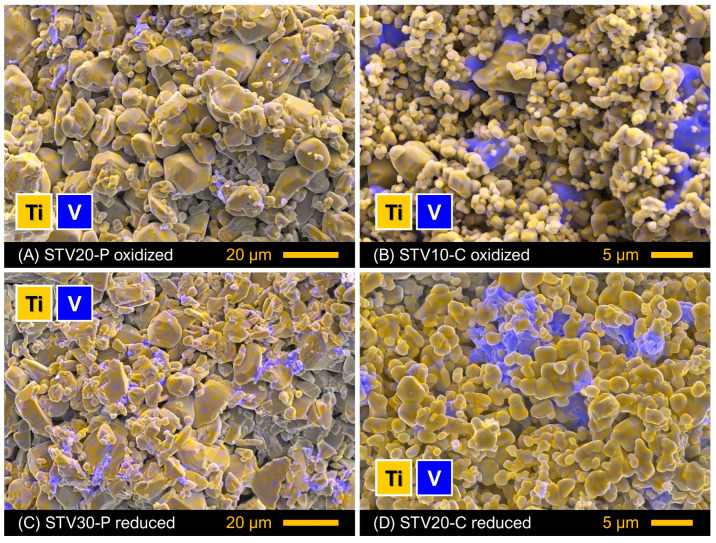
Examples of SEM images with overlaid EDS elemental mapping obtained from the fractured surface of (**A**,**B**) as-prepared ceramics and (**C**,**D**) reduced samples.

At the same time, EDS analysis revealed the presence of V-rich inclusions in all as-prepared STV-P samples; a representative example of an SEM/EDS image is shown in Figure 3A. These observations indicate that, in fact, STV-P samples comprised secondary phases of the Sr-V-O system undetected by the XRD, either due to a small fraction or an amorphous state. Note that the processing conditions were close to the melting point of Sr_2_V_2_O_7_ pyrovanadate (1125–1160 °C [44,45,46]). The presence of Sr-V-O impurities also implies that the actual vanadium content in the titanium sublattice of prepared SrTi_1−*y*_V*_y_*O_3±δ_ perovskites is somewhat below the nominal.

The lower processing temperature compared to the STV-P series resulted in a smaller average grain size of STV-C samples (Figure 2B). The size of particles varied between 0.5–1.0 and 5–7 µm. Sr-V-O components were found to exist mainly as agglomerates of 3.5–5.5 µm in size (Figure 3B). The STV-C composites had even lower densities with respect to STV-P ceramics (Table 1), which may indicate comparable or higher porosity. Note that the theoretical density of Sr_3_(VO_4_)_2_ (4.47 g/cm^3^) and β-Sr_2_V_2_O_7_ (4.05 g/cm^3^) (see ICDD PDF no. 01-071-1593 and 01-081-1844) is lower compared to SrTiO_3_ (~5.13 g/cm^3^).

### 3.2. Phase Changes on Reduction

**Figure 4:**
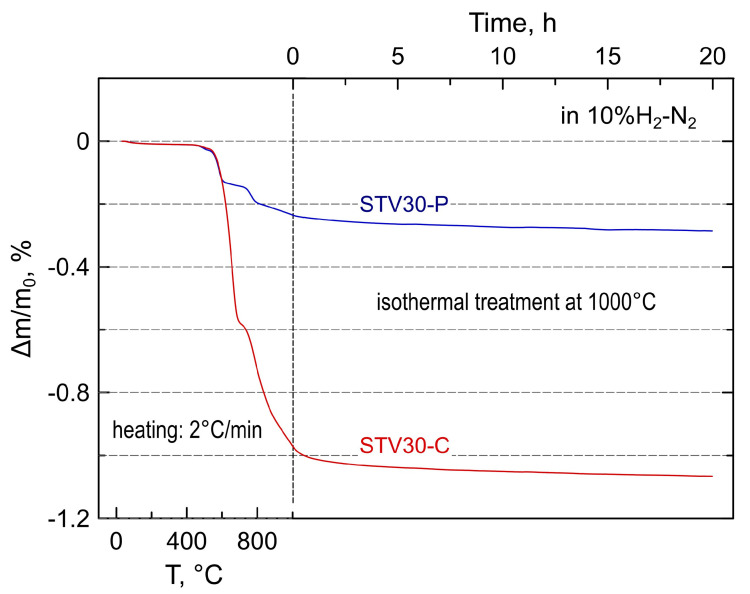
Relative weight loss of STV30 samples on reduction in a 10%H_2_-N_2_ mixture flow. The procedure included heating at 2 °C/min to 1000 °C followed by isothermal treatment at this temperature for 20 h.

Figure 4 shows the representative thermogravimetric data for powdered STV30-P and STV30-C samples recorded during the reduction in the 10%H_2_-N_2_ atmosphere. The shape of the TGA curves on heating suggests that reduction involves several processes that may include phase and structural transformations and changes in the oxidation state of vanadium cations in the perovskite-type Sr(Ti,V)O_3±δ_ lattice. The partial reduction of titanium cations may also occur to some extent, but the contribution of this process to overall weight changes is expected to be negligible. 

The TGA curve of the STV-C sample (Figure 4) qualitatively resembles the corresponding curve reported for Sr_2_V_2_O_7_ under similar conditions, with a characteristic inflection at 690–730 °C [26,27]. It has been suggested that the reductive transformation of Sr_2_V_2_O_7_ pyrovanadate into SrVO_3−δ_ perovskite occurs via the 5Sr_3_(VO_4_)_2_ + SrV_6_O_11_ intermediate, and the inflection in the thermogravimetric curve corresponds to the complete transformation of pyrovanadate into the intermediate mixture and the onset of perovskite phase [26]. While this first step occurs comparatively fast, the kinetics of the reduction of Sr_3_(VO_4_)_2_ is slower, and a higher temperature and a longer time are required for the complete conversion of orthovanadate into perovskite-type SrVO_3−δ_. This is reflected by a slow drift in sample weights during the isothermal reduction step at 1000 °C (Figure 4).

**Figure 5:**
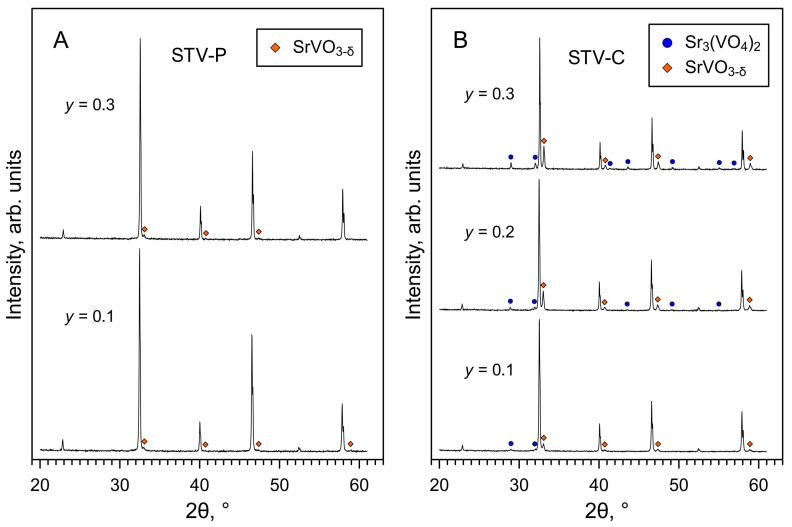
XRD patterns of (**A**) STV-P and (**B**) STV-C samples reduced in a 10%H_2_-N_2_ mixture flow at 1000 °C for 20 h. The reflections of the SrVO_3−δ_ and Sr_3_(VO_4_)_2_ phases are marked according to ICDD PDF no. 01-081-0119 and 01-081-1844, respectively.

The XRD inspections of the reduced STV-P samples revealed the presence of a small fraction of a second perovskite phase in addition to the main SrTiO_3_-based solid solution (Figure 5A). This phase was identified as perovskite-like SrVO_3−δ_. This indicates that the Sr-V-O inclusions in as-prepared STV-P samples undetected by the XRD transformed into SrVO_3−δ_ on reduction (although traces of Sr_3_(VO_4_)_2_ cannot be completely excluded). The reduced STV-C samples consisted of three detectable phases: perovskite-type SrTiO_3_, SrVO_3−δ_ and unconverted Sr_3_(VO_4_)_2_ orthovanadate (Figure 5B). The fraction of SrVO_3−δ_ increased with the total vanadium content, while the fraction of residual strontium orthovanadate showed the opposite trend. Reduction had no noticeable effect on the microstructure of STV-P and STV-C samples (Figure 3C,D), except that the surface of Sr-V-O agglomerates, which was smooth in oxidized samples, became etched after reduction as a result of phase transformation. 

The weight changes during the reduction of the STV-C sample (Figure 4) can be assigned to a massive reduction of V^5+^ to V^4+^ or mixed V^4+/3+^ state in the course of transformation of Sr_2_V_2_O_7_ and Sr_3_(VO_4_)_2_ into SrVO_3−δ_. Note that SrVO_3−δ_ exhibits variable oxygen nonstoichiometry under reducing conditions, which tends to δ~0.1 at 1000 °C in a 10%H_2_-N_2_ atmosphere [26,27,28]; this corresponds to the average oxidation state of vanadium cations of ~3.8+. The weight loss during the reduction of the STV-P sample was ~5 times lower (Figure 4). Assuming that all vanadium is in a 4+ state after heating to 1000 °C in a 10%H_2_-N_2_ atmosphere, rough estimations yield the average oxidation state of vanadium of ~4.18+ in the oxidized as-prepared sample. This observation implies that the formation of SrTi_1−*y*_V*_y_*O_3±δ_ perovskite under oxidizing conditions follows the first scenario (see discussion above): vanadium is forced into a 4+ state to incorporate into the titanium sublattice even under oxidizing conditions. The average oxidation state slightly higher than 4+ probably mainly originates from V^5+^ in the residual secondary phases. Earlier, Park et al. concluded, based on X-ray photoelectron spectroscopy results, that V^4+^ predominates over V^5+^ in SrTi_0.8_V_0.2_O_3_ thin films prepared under an ambient atmosphere [38].

### 3.3. Thermal Expansion and Dimensional Changes

**Figure 6:**
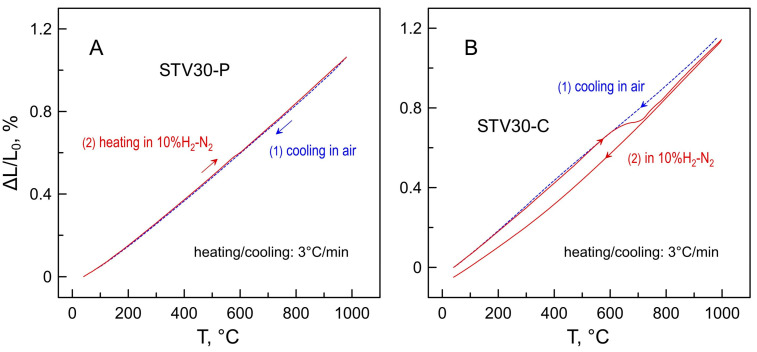
Dilatometric curves of (**A**) STV30-P and (**B**) STV30-C ceramic samples in air and in a 10%H_2_-N_2_ atmosphere. The thermal cycle in air was followed by a cycle in reducing atmosphere.

Figure 6 depicts the representative dilatometric data obtained in air and in a 10%H_2_-N_2_ atmosphere. Both the STV-P and STV-C samples exhibit smooth, nearly linear thermal expansion in air. The average linear thermal expansion coefficients in air vary in a narrow range of 11.1–12.1 ppm/K at 30–1000 °C (Table 2). The thermomechanical behavior of the prepared samples is defined by the properties of the SrTiO_3_ perovskite phase with TEC = 11.7 ppm/K. Note that Sr_2_V_2_O_7_ and Sr_3_(VO_4_)_2_ ceramics were reported to exhibit slightly higher TECs in air, 15.0 and 14.3 ppm/K, respectively [26]. The TECs of STV-P and STV-C are also close to that of common solid electrolyte ceramics including yttria-stabilized zirconia, doped ceria and lanthanum gallate-based perovskites (Table 2). This ensures good thermomechanical compatibility between solid electrolytes and STV-based electrode layers.

The dilatometric curves of oxidized STV-P samples recorded on heating in a reducing 10%H_2_-N_2_ atmosphere are nearly identical to the dilatometric data obtained in air (Figure 6A). Once again, this is determined by the properties and high redox stability of the SrTiO_3_ perovskite lattice. Only a minor inflection associated with the reduction process was detected in the dilatometric curves at ~580–600 °C during the first heating. 

The dimensional changes caused by the reduction process were more evident in the dilatometric data of STV-C ceramic samples (Figure 6B). The inflection at 630–720 °C can be clearly seen in the dilatometric curve during the first heating in the reducing atmosphere. The contraction is comparatively small, ~0.09% in linear dimensions, and corresponds to the first step of the reductive phase transformation evidenced by the thermogravimetric analysis (Figure 4). Note that estimations based on the structural data and the results of the dilatometric experiments indicate substantial dimensional changes on redox transformations between oxidized and reduced strontium vanadates [25,26]. In particular, the comparison of unit cell volumes implies that the complete transformation of tetragonal β-Sr_2_V_2_O_7_ pyrovanadate (V_UC_ = 1276.18 Å^3^, *z* = 8, ICDD PDF no. 01-071-1593) into cubic SrVO_3−δ_ (V_UC_ = 56.66 Å^3^, *z* = 1, ICDD PDF no. 01-081-0119) should result in volume shrinkage by ~29% (or ~11% in linear dimensions). In the case of porous STV-C samples, the fraction of Sr_2_V_2_O_7_ is comparatively small, and reduction-induced dimensional changes are partly accommodated by the volume of pores. This ensures only a minor shrinkage of composite samples caused by reduction. After the first heating cycle, the reduced STV-C ceramics showed smooth moderate dimensional changes on thermal cycling in a 10%H_2_-N_2_ atmosphere.

### 3.4. Electrical Conductivity

**Figure 7:**
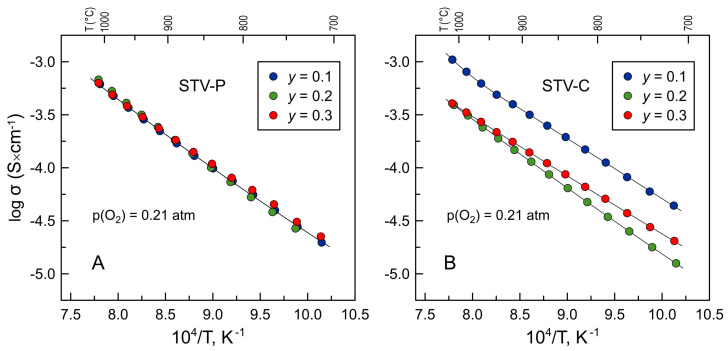
Total electrical conductivity of (**A**) STV-P and (**B**) STV-C ceramics in air.

Figure 7 shows the data on the electrical conductivity of as-prepared materials measured in air at 750–1000 °C. All ceramics exhibit semiconducting behavior and comparatively low values of electrical conductivity, in the range (1–4) × 10^−4^ S/cm at 900 °C, partly due to the high porosity of the samples. The electrical conductivity of STV-P ceramics was essentially independent of the composition. On the contrary, the total conductivity of STV-C composites varies with nominal vanadium content and decreases in the sequence, σ_STV10_ > σ_STV30_ > σ_STV20_. This seems to reflect an interplay between the fractions and conductivity of individual phases and the porosity of the samples. The values of the electrical conductivity of “undoped” polycrystalline SrTiO_3_ ceramics reported in the literature depend on the purity and fabrication conditions and may reach 7 × 10^−3^ S/cm at 900 °C in air [16]. Polycrystalline Sr_2_V_2_O_7_ and Sr_3_(VO_4_)_2_ ceramics exhibit lower conductivity under these conditions, ~4 × 10^−4^ and 1 × 10^−4^ S/cm, respectively [26,51]. Thus, the electrical conductivity of STV-C composites initially decreases with increasing fractions of low-conducting Sr-V-O phases but then slightly increases for the STV30-C sample due to reduced porosity (as follows from the values of density, Table 1). 

**Figure 8:**
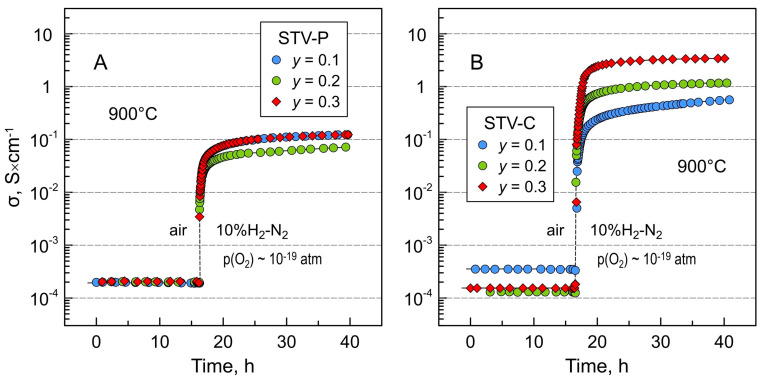
Relaxation of electrical conductivity of (**A**) STV-P and (**B**) STV-C ceramics on reduction at 900 °C after switching from oxidizing (air) to a reducing (10%H_2_-N_2_) atmosphere.

The isothermal reduction in the 10%H_2_-N_2_ atmosphere at 900 °C results in ~2.5 orders of magnitude increase in the electrical conductivity of porous STV-P ceramics (Figure 8A). An increase in conductivity on reduction can be attributed to the partial reduction of titanium and vanadium cations with the generation of n-type electronic charge carriers:

2Ti^4+^ + O^2−^ → 2Ti^3+^ + 0.5O_2_, (1)

2V^4+^ + O^2−^ → 2V^3+^ + 0.5O_2_, (2)

where Ti^3+^ and V^3+^ are equivalent to electrons localized on titanium and vanadium cations, respectively. These equations can be rewritten as:

O^2−^ → 2e^−^ + 0.5O_2_, (3)

Electronic conductivity in the reduced STV-P perovskites is likely to occur via electron hopping between Ti^4+^/Ti^3+^ and V^4+^/V^3+^ redox pairs. The defect chemistry and electrical properties of reduced Sr(Ti,V)O_3−δ_ are discussed in detail in [28]. In addition to the increase in intrinsic electrical conductivity of the perovskite phase, the enhancement in electrical properties of STV-P samples on reduction is also likely to be partly contributed by the transformation of residual Sr-V-O impurities into high-conducting SrVO_3−δ_ perovskite. After ~24 h of reduction, the conductivity reaches (0.7–1.2) × 10^−1^ S/cm and continues to grow slowly, but substantial further improvement is not expected. These values are noticeably lower compared to the conductivity of the single-phase SrTi_1−*y*_V*_y_*O_3−δ_ counterparts prepared under reducing conditions (Table 3), particularly in the case of *y* = 0.2 and 0.3, despite comparable relative density [28]. 

STV-C composite ceramics showed a more significant increase in electrical conductivity on reduction—by 3–4 orders of magnitude with respect to the level of σ under air (Figure 8B). In this case, a sharp improvement in electrical properties on reduction is associated mainly with the transformation of insulating Sr_2_V_2_O_7_ and Sr_3_(VO_4_)_2_ phases into SrVO_3−δ_ perovskite, which has approximately seven orders of magnitude higher conductivity at this temperature. Electrical transport in the reduced STV-C composites can be assumed to occur through percolating SrVO_3−δ_ particles and agglomerates and gradually increases with the increasing nominal vanadium content and the fraction of the SrVO_3−δ_ component (Figure 5B). Furthermore, the values of electrical conductivity of STV-C samples after reduction for ~24 h are similar to or even exceed the corresponding values reported for single-phase SrTi_1−*y*_V*_y_*O_3−δ_ (*y* = 0.1–0.3) perovskites (Table 3). The σ of porous reduced STV30-C reaches 3.4 S/cm, which is not optimal but may be acceptable for a solid oxide cell electrode material. In the case of the well-distributed current collection, the target conductivity for porous anode structures is set to >1 S/cm (or >10 S/cm for intrinsic material properties in a dense form) [52], although there are indications that reasonable electrode performance can be obtained for mixed-conducting anode materials with adequate electronic conductivity of ~0.1 S/cm [53].

## 4. Conclusions

The thermal processing of precursors with nominal composition SrTi_1−*y*_V*_y_*O_z_ (*y* = 0.1, 0.2, 0.3) at temperatures not exceeding 1100 °C yields composite ceramics comprising perovskite-type SrTiO_3_, pyrovanadate Sr_2_V_2_O_7_ and orthovanadate Sr_3_(VO_4_)_2_ phases. The fractions of vanadate phases increase with nominal vanadium content, and orthovanadate dominates over pyrovanadate. Increasing firing temperatures to 1250–1440 °C enables the formation of SrTi_1−*y*_V*_y_*O_3±δ_ perovskites. While the XRD results suggested that these ceramics are phase-pure, the SEM/EDS analysis revealed the co-existence of Sr-V-O precipitates, indicating that vanadium content in the perovskite lattice is lower than nominal. The results of the thermogravimetric analysis suggest that vanadium substitutes into titanium sublattice predominantly as V^4+^ even under oxidizing conditions at elevated temperatures. 

Both perovskite and composite materials exhibit moderate thermal expansion coefficients in air, 11.1–12.1 ppm/K at 30–1000 °C. Reduction results in negligible dimensional changes in perovskite samples and only minor contractions of composite ceramics (<0.1% in linear dimensions during the first heating). While as-prepared porous ceramic samples exhibit low electrical conductivity in air in the order of 10^−4^ S/cm at 900 °C, reduction in 10%H_2_-N_2_ at 900 °C results in an increase in conductivity by several orders of magnitude. The electrical conductivity of perovskite samples remains comparatively low after reduction, ~0.1 S/cm at 900 °C. On the contrary, the transformation of oxidized vanadate phases in composite samples into high-conductive perovskite-like SrVO_3−δ_ on reduction results in substantially higher conductivity, which increases with nominal vanadium content and reaches 3.4 S/cm at 900 °C for porous ceramics with nominal SrTi_0.7_V_0.3_O_z_ composition. 

The processing route employing multi-component oxidized Sr(Ti,V)O*_z_* precursors appears to be a preferential approach enabling the preparation of composite anodes, where SrTiO_3_ acts as a redox-stable structural element and the SrVO_3_ phase is responsible for electrical performance. In follow-up work, further improvements are expected to be achieved through the modification of the SrTiO_3_ component via donor-type doping and the optimization of the processing route and microstructure to facilitate the reduction process of oxidized precursors and attain a more uniform distribution and percolation of the SrVO_3_ phase. 

## Figures and Tables

**Table 1:** Parameters of cubic perovskite lattice and density of as-prepared ceramic samples.

Nominal *y*	STV-P				STV-C
	*a*, Å	Density, g/cm^3^	Relative Density, % ^1^		Density, g/cm^3^
0.1	3.9059(1)	3.20	63		2.31
0.2	3.9047(1)	3.30	64		2.38
0.3	3.9042(1)	3.29	64		2.81

^1^ Theoretical density of STV-P oxides was estimated assuming nominal cation composition and neglecting possible oxygen nonstoichiometry.

**Table 2:** Average thermal expansion coefficients ($\bar{\alpha }$) calculated from the dilatometric data in air.

Composition	T Range, °C	$\bar{\alpha }$ × 10^6^, K^−1^
STV10-P	30–1000	11.2
STV20-P	30–1000	11.1
STV30-P	30–1000	11.1
STV30-C	30–1000	12.1
SrTiO_3_ [16]	30–1100	11.7
8YSZ, (ZrO_2_)_0.92_(Y_2_O_3_)_0.08_ [50]	30–1000	10.9
CGO20, Ce_0.8_Gd_0.2_O_2−δ_ [50]	30–1000	12.7
LSGM, La_0.9_Sr_0.1_Ga_0.8_Mg_0.2_O_3−δ_ [50]	30–1000	11.4

**Table 3:** Electrical conductivity of nominal SrTi_1−*y*_V*_y_*O*_z_* after reduction for 24 h in a 10%H_2_-N_2_ atmosphere at 900 °C.

Nominal *y*	σ, S/cm (900 °C, p(O_2_)~10^−20^ atm)		
	STV-P	STV-C	SrTi_1−*y*_V*_y_*O_3−δ_ [28] ^1^
0.1	0.12	0.58	0.096
0.2	0.072	1.2	0.56
0.3	0.12	3.4	3.3

^1^ Single-phase perovskite ceramics synthesized and sintered under reducing conditions at 1500 °C.

## Data Availability

Data are contained within the article and available from the corresponding author upon reasonable request.
